# Project ACCLAIM: Intervention Effect on Community Knowledge, Attitudes and Beliefs of Maternal and Child Health and HIV/AIDS in Eswatini, Uganda and Zimbabwe

**DOI:** 10.1007/s10461-021-03202-2

**Published:** 2021-03-04

**Authors:** N. Bandopadhay, G. B. Woelk, M. P. Kieffer, D. Mpofu, the Project ACCLAIM Study Group, Godfrey Woelk, Mary Pat Kieffer, Dephine Mpofu, Caspian Chouraya, Kwashie Kudiabor, Philisiwe Khumalo, Edward Bitarakwate, Eliab Natumanya Kajungu, Agnes Mahomva, Reuben Musarandega

**Affiliations:** 1 Milken Institute School of Public Health George Washington University, Washington DC, 20036 USA; 2 Elizabeth Glaser Pediatric AIDS Foundation, 1140 Connecticut Avenue NW, Suite 200, Washington DC, 20036 USA; 3 Elizabeth Glaser Pediatric AIDS Foundation, Mbabane, Eswatini; 4 Elizabeth Glaser Pediatric AIDS Foundation, Mbarara, Uganda; 5 Elizabeth Glaser Pediatric AIDS Foundation, Harare, Zimbabwe

**Keywords:** HIV/AIDS, Maternal and child health, PMTCT, KAB, HIV stigma, Gender, Community interventions

## Abstract

The ACCLAIM Study aimed to assess the effect of a package of community interventions on the demand for, uptake of, and retention of HIV-positive pregnant/postpartum women in maternal and child health (MCH) and prevention of mother-to-child HIV transmission (PMTCT) services. The study occurred from 2013 to 2015 in Eswatini, Uganda, and Zimbabwe. The three interventions were: (1) a social learning and action component for community leaders, (2) community days, and (3) peer discussion groups. Household cross-sectional surveys on community members’ MCH and PMTCT knowledge, attitudes, and beliefs were analyzed pre- and post-intervention, using MCH, HIV stigma, and gender-equitable men (GEM) indicators. We used t-tests to measure the significance of mean pre- vs. post-intervention score changes stratified by gender within each intervention arm and generalized linear models to compare mean score changes of the cumulative intervention arms with the community leaders-only intervention. Response rates were over 85% for both surveys for men and women, with a total of 3337 pre-intervention and 3162 post-intervention responses. The combined package of three interventions demonstrated a significantly greater increase in MCH scores for both women (diff = 1.34, p ≤ 0.001) and men (diff = 2.03, p < 0.001). The arms that included interventions for both community leader engagement and community days (arms 2 and 3)led to a greater increase in mean GEM scores compared to the community leader engagement intervention alone (arm 1), for both women (diff = 1.32, p = 0.002) and men (diff = 1.37, p = 0.004). Our findings suggest that a package of community interventions may be most effective in increasing community MCH/HIV knowledge and improving gender-equitable norms.

## Introduction

Innovative approaches are needed to eliminate mother-to-child HIV transmission (MTCT) in high-burden (priority) countries. With the adoption of universal treatment for people living with HIV in most countries, including for pregnant women, 6-week MTCT rates have fallen dramatically in the 21 priority countries, declining from 28% [25–30%] in 2009 to 14% [12–16%] in 2014 [1]. However, 160,000 children were newly infected with HIV in 2018 [2]. Significant barriers remain that are not specific to HIV and that interfere with uptake of HIV treatment among pregnant women. These barriers include inequitable gender norms, HIV-related stigma, and inadequate mother and child health (MCH) knowledge [3–6]. Interventions to address these barriers have included community health promotion and education activities, and engagement strategies [4, 7]. However, many of these approaches focus on increasing knowledge, with insufficient attention paid to attitudes and norms; which is necessary to enable sustainable behavior change at the community level [8]. In addition, barriers to prevention of mother-to-child HIV transmission (PMTCT) services are often interlinked; in that interventions which target both community and individual levels, may be more effective than interventions focused on one level. There are few examples of combined community-level interventions for PMTCT outcomes [9]. We tested an innovative community intervention package which included a social learning and action component (community leader engagement), community days (with community dialogues around selected MCH and PMTCT topics), and peer discussion groups, on the demand for, uptake of, and retention of HIV-positive pregnant/postpartum women in MCH/PMTCT services in Eswatini (formerly Swaziland), Uganda, and Zimbabwe from 2013 to 2015.

In this paper, we report on the effect of three interventions on MCH and PMTCT knowledge, attitudes, and beliefs (KAB) among community members in the study areas. This was a secondary analysis of the trial data focused on the community effects of the interventions. We assessed gender-equitable norms, HIV-related stigma attitudes, and knowledge on maternal, neonatal, and childcare. We hypothesized that the three interventions together (community leader engagement, community days, and peer discussion groups) would result in the most significant increase in MCH beliefs and gender-equitable norms, as well as the most significant decrease in HIV stigma, when compared to the community leader engagement intervention alone for both women and men, when comparing pre- and post-intervention data.

## Methods

### Study Design and Population

**Fig. 1:**
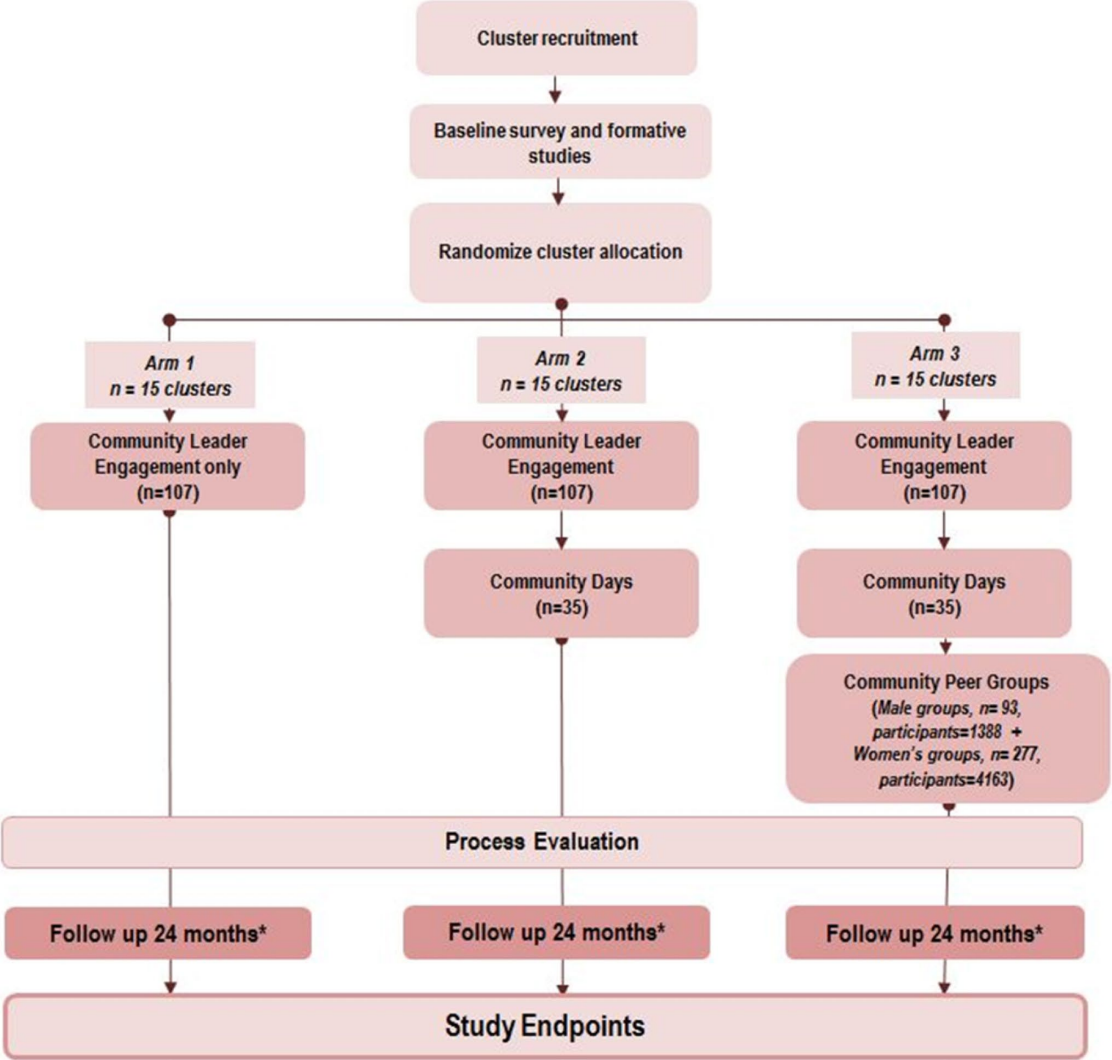
Trial design. Reprinted from “Evaluating the effectiveness of selected community-level interventions on key maternal, child health, and prevention of mother-to-child transmission of HIV outcomes in three countries (the ACCLAIM project): a study protocol for a randomized controlled trial,” by Woelk, G.B., Kieffer, M.P., Walker, D., Mpofu, D., Machekano, R., and the Project ACCLAIM study group. *Trials, 17(88)* 2016, p.5

The methodology of this multi-country, multi-component, three-arm randomized trial has been reported elsewhere [10]. In brief, in each of the three countries, three subunits (regions or districts) were identified, each consisting of 15 clusters (facilities) (Fig. 1). The regions or districts were then randomly allocated, one to each study arm. Arm 1 included the community leader engagement intervention, which involved identifying and training formal and informal community leaders to have dialogues with their community about MCH, HIV care, and PMTCT. Arm 2 included the community leader engagement intervention plus a community days intervention (i.e., community health fairs that combined service provision such as HIV testing, blood pressure screening, etc., with community dialogues about HIV issues, PMTCT, and HIV testing. Arm 3 included the community leader engagement intervention, the community days intervention, plus men’s and women’s peer discussion groups, where peer-led discussions provided information and education on MCH and PMTCT. The women’s peer groups were held with women attending antenatal care (ANC); the men’s groups included, but were not limited to, partners of the women attending ANC.

There was no control group, as there was already a high level of background community programming in the form of national PMTCT programs in study countries. The study team concluded therefore, that a true control group would be infeasible and unethical [6, 10].

In each country, Elizabeth Glaser Pediatric AIDS Foundation (EGPAF)-supported regions with no recent history of intensified community-level PMTCT interventions and low research activity but moderate to high maternal HIV yield were identified as potential intervention areas. The three subunits (one for each of the three study arms) were identified corresponding to the administrative structures (that is, regions or districts) existing in that country, also took into account the ability to implement interventions, with the support of the appropriate health authorities. In each of these districts/regions, five clusters (with a cluster defined as the lowest level of health facility that implements PMTCT services, together with its population catchment area [populations 7300–27,500]) were identified. Criteria for potential selection included recording at least 14 HIV-positive pregnant women in the most recent year, the smallest catchment population size, and catchment area completely within the district/region with no overlap, and mix of facility type. Referral facilities and urban facilities were excluded. All clusters meeting these criteria were selected.

In order to assess potential changes in MCH knowledge, attitudes, and beliefs, we undertook pre- and post-intervention KAB cross-sectional surveys among women and men aged 18–60 years in randomly selected households in the selected clusters in each country. With the cross-sectional design, different households were likely to be selected pre- and post-intervention. One participant per household was selected. If there was more than one eligible participant per household, research assistants randomly selected a participant through a process involving the listing of all eligible participants. The randomization was based on sampling frames drawn from census lists and augmented by household lists or maps held by community leaders and/or the relevant administrative offices. The surveys were enumerated by trained research assistants with appropriate supervision and the data were captured directly into an EPIINFO v7.1 database on laptop computers in the field. The survey was translated into the local languages (Runyankole, SiSwati, and Shona) and pre-tested.

### Survey Questions and Scoring

The survey questions (statements) were drawn from items of validated scales and indicator compendiums of the *Gender Equitable Men* (*GEM*) *Scale* [11], the *Tanzania Stigma Indicator and Community Baseline* (*Individual Questionnaire*) [12], the *World Bank Social Capital Assessment Tool* (*A*-*SCAT*) [13], and the *Rapid CATCH* + *2009 Maternal and Neonatal Care Module* [14]. The main outcomes for this analysis are MCH beliefs scores, (from the *Rapid CATCH* + *2009 Maternal and Neonatal Care Module),* HIV stigma scores (from the *Tanzania Stigma Indicator and Community Baseline* (*Individual Questionnaire*), and the GEM norms scores. Table 1 shows an example of some of these questions.

**Table 1:** Survey questions used for outcome scores

Questions used for MCH beliefs scores
Vaginal bleeding during pregnancy is caused by pregnant mother working too hard
Maternal or infant death is caused by evil spirits or breaking taboos
I believe in my ability to plan for the safe delivery for myself and my child
Community members would agree that ensuring healthy outcomes for every mother and child in the village is a priority for this community
There are skilled community members who would be willing to work together to solve the problems that prevent healthy outcomes for every mother and child in this community
Questions used for HIV stigma scores
Most people in this community would want to keep it a secret if a member of their family had HIV
If a health worker is HIV-positive, I don’t think they should be allowed to treat patients
HIV/AIDS is the result of sinning
People with HIV/AIDS should be allowed to fully participate in social events in this community
People living with HIV/AIDS should not be ashamed
Questions used for gender-equitable norms scores
It is the man who decides what type of sex to have
It is a woman’s responsibility to avoid getting pregnant
A man should have the final word about decisions in his home
There are times when a woman deserves to be beaten
Men in the community see it as unmanly to be involved in issues related to their partner’s pregnancy and childbirth

The three outcome scores were numeric, and these outcomes were scored according to the scheme below:

MCH beliefs score: Responses to questions regarding MCH beliefs were scored based on a scale of 0–4; from “strongly agree” (4) to “strongly disagree” (1), and “neither agree nor disagree” (0) for true statements. False statements were reverse scored; “strongly agree” (1), to “strongly disagree” (4). Scores from each question were added up for each individual. A higher score indicated a better community understanding of MCH issues and a greater confidence in the community to address MCH-related HIV issues. For the pre-intervention surveys, Cronbach’s alpha scores ranged from 0.31 to 0.48 across the three countries. For the post-intervention surveys, these scores ranged from 0.40 to 0.52.

HIV stigma score: Responses to HIV-related stigma questions were scored 4-1; “strongly agree” (4) to “strongly disagree” (1). Scores for each question were added for each individual, with a lower composite score reflective of less HIV-related stigma. Cronbach’s alpha scores ranged from 0.38 to 0.47 across the three countries for the pre-intervention surveys. For the post-intervention surveys, these scores ranged from 0.27 to 0.60. Measuring HIV stigma was important, as other studies pointed out that HIV stigma is a key barrier to HIV prevention and treatment efforts [15].

Gender-equitable norms score: Scoring for the gender-equitable norms section was based on the GEM Scale [11]. This section contained questions with statements that reflect gender inequitable norms. An “Agree” response was given a score of 1, “Partially agree” a score of 2, and “Disagree” a score of 3. Scores for each question were summed to a composite score for each individual. A higher score was indicative of a higher degree of gender-equitable norms. Cronbach’s alpha scores for the pre-intervention surveys across the three countries were 0.60–0.73, and 0.64–0.75 for the post-intervention surveys.

Out of several outcomes, these three scores were chosen because previous studies have failed to address community MCH support, HIV stigma within the community, and gender norms that affect women’s participation in PMTCT programs [10]. In addition to these outcomes, the questionnaire consisted of items in the following areas: problems in accessing health care; individual MNCH knowledge, attitudes, perceptions and barriers; sexually transmitted infections; HIV knowledge; HIV testing and disclosure; PMTCT knowledge; pregnancy and postnatal care (for women), GEM-equitable norms; household decision-making; contraception; and social capital (groups and networks).

We selected the three outcomes, as they were the most discriminant across the study arms and countries (data not shown). In addition, the outcome measures selected represent indicators of barriers and facilitators to PMTCT services. Factors that affect pregnancy outcomes overall are relevant for HIV-positive pregnant women. For example, the measure of “Vaginal bleeding during pregnancy is caused by pregnant mother working too hard” is an indicator of knowledge that working burden (working too hard) during pregnancy could result in a negative outcome of vaginal bleeding. The statement that maternal or infant death being caused by evil spirit or breaking taboos is a measure of superstition that is relevant for PMTCT as a belief that for example, death of an infant due to HIV could be because of evil spirits. The other MCH statements reflect a degree of self-efficacy and community cohesion; that a mother is able to act to have a successful birth outcome, and that the community members can work together to have healthy outcomes for mother and children, including PMTCT. Stigma is, of course, is a barrier to PMTCT, and so is gender inequity, where husbands for example, may be less supportive of their wives in pregnancy (especially for PMTCT), or unwilling to financially support women’s attendance at ANC. These three outcomes were measured for both women and men, as previous studies did not assess men’s attitudes in the community towards MCH support, which can affect women’s health seeking behaviors and participation in PMTCT programs.

### Analysis

We analyzed data by country and gender from the KAB community pre- and post-intervention surveys. Women completed 1783 pre-intervention surveys and 1807 post-intervention surveys. Men completed 1554 pre-intervention surveys and 1355 post-intervention surveys. We tested the distribution of scores for MCH beliefs, HIV stigma, and GEM for normality. Mean MCH beliefs scores, HIV stigma scores, and GEM scores for each study arm were calculated from the pre-intervention surveys and the post-intervention surveys. T-tests were used to assess the difference between the mean pre-intervention scores and the mean post-intervention scores.

Although randomization was used for intervention assignment, descriptive data were compared for potential confounders across groups. The main potential confounders included age, highest level of education, number of years in the community, whether one is living away from home, source of income, and marital status.

We undertook a differences-in-differences analysis, comparing arms 2 and 3 against arm 1. We used generalized linear models with contrast analysis to test whether the post- vs. pre-intervention mean score difference was significantly different in arms 2 and 3, compared to arm 1. A total of 6479 records were available for analysis. All analyses were undertaken using SAS 9.4.

## Results

Response rates were over 85% for both surveys and men and women. Table 2 presents the demographic characteristics of the study participants.

**Table 2:** Demographic characteristics of survey participants by country

	Women				Men			
	Zimbabwe n = 658	Uganda n = 556	Eswatini n = 549	Total n = 1763	Zimbabwe n = 421	Uganda n = 584	Eswatini n = 552	Total n = 1557
	n (%)	n (%)	n (%)	n (%)	n (%)	n (%)	n (%)	n (%)
Age (mean, SD)	36.4 (12.1)	35.8 (11.0)	34.7 (11.2)		35.2 (11.5)	39.8 (11.3)	33.9 (12.3)	
Age group (years)								
< 19	32 (4.9)	17 (3.1)	19 (3.5)	68 (3.9)	23 (5.5)	18 (3.1)	30 (5.4)	71 (4.5)
20–29	196 (29.8)	164 (29.9)	200 (36.4)	560 (31.8)	136 (32.3)	104 (17.8)	225 (40.8)	465 (29.9)
30–39	185 (28.1)	173 (31.1)	150 (27.3)	508 (28.8)	124 (29.4)	160 (27.4)	129 (23.4)	413 (26.5)
40–49	114 (17.3)	122 (21.9)	105 (19.1)	341 (19.3)	77 (18.3)	171 (29.3)	77 (13.9)	325 (20.9)
50 +	131 (19.9)	80 (14.4)	75 (13.7)	286 (16.2)	61 (14.5)	131 (22.4)	91 (16.5)	283 (18.2)
Education								
Primary or less	224 (34.0)	446 (80.2)	238 (43.4)	908 (51.5)	90 (21.4)	380 (65.3)	228 (41.3)	698 (44.9)
Secondary or more	434 (66.0)	110 (19.8)	310 (56.6)	854 (48.5)	330 (78.6)	202 (34.7)	324 (58.7)	856 (55.1)
Missing			1	1	1	2		3
Religion								
Roman Catholic	58 (8.8)	157 (28.8)	12 (2.2)	227 (12.9)	59 (14.0)	177 (30.4)	15 (2.7)	251 (16.2)
Protestant	178 (27.1)	341 (62.5)	62 (11.3)	581 (33.1)	99 (23.6)	351 (60.2)	65 (11.8)	515 (33.1)
Pentecostal	89 (13.6)	26 (4.8)	220 (40.1)	335 (19.1)	38 (9.0)	36 (6.2)	143 (26.0)	217 (14.0)
Apostolic Sect	299 (45.5)	3 (0.5)	227 (41.4)	529 (30.2)	120 (28.6)	2 (0.3)	239 (43.4)	361 (23.2)
Other	7 (1.1)	19 (3.4)	9 (1.6)	35 (2.0)	8 (1.9)	17 (2.9)	15 (2.7)	40 (2.6)
None	27 (4.1)	0	19 (3.4)	46 (2.6)	96 (22.9)	0	74 (13.4)	170 (10.9)
Missing		10		10	1	1	1	3
No. of years in community								
< 1	31 (4.7)	41 (7.4)	15 (2.8)	87 (5.0)	25 (5.9)	2 (0.3)	10 (1.8)	37 (2.4)
1–5	127 (19.3)	44 (7.9)	87 (16.1)	258 (14.7)	50 (11.9)	9 (1.4)	44 (8.1)	102 (6.6)
> 5	500 (76.0)	471 (84.7)	437 (81.1)	1408 (80.3)	346 (82.2)	571 (98.3)	489 (90.1)	1406 (91.0)
Missing			10	10				
Away from home > 1 month at a time in the past 12 months								
Yes	208 (31.6)	79 (14.2)	116 (21.1)	403 (22.9)	165 (39.2)	121 (20.9)	163 (29.5)	449 (28.9)
No	450 (68.4)	477 (85.9)	433 (78.9)	1360 (77.1)	256 (60.8)	459 (79.1)	389 (70.5)	1104 (71.1)
Marital status								
Married/living together	489 (74.3)	413 (74.7)	318 (58.3)	1220 (69.5)	292 (69.5)	504 (86.3)	235 (42.8)	1031 (66.4)
Never married	30 (4.5)	35 (6.3)	183 (33.6)	248 (14.1)	100 (23.8)	61 (10.5)	293 (53.4)	454 (29.2)
Divorced/separated	57 (8.7)	27 (4.9)	8 (1.5)	92 (5.2)	19 (4.5)	14 (2.4)	10 (1.8)	43 (2.8)
Widowed	82 (12.5)	78 (14.1)	36 (6.6)	196 (11.2)	9 (2.1)	5 (0.9)	11 (2.0)	25 (1.6)
Missing		3	4	7	1		3	4
No. in household (mean, median)	5.34 (2.46)	5.87 (6.00)	6.50 (6.00)		5.0 (2.4)	6.3 (6.0)	7.1 (7.0)	
Main source of income								
Farming	319 (48.6)	431 (77.5)	135 (24.6)	885 (50.2)	198 (47.1)	332 (57.0)	168 (30.5)	698 (44.9)
Formal employ	26 (3.9)	28 (5.0)	55 (10.0)	109 (6.2)	54 (12.9)	78 (13.4)	70 (12.7)	202 (13.0)
Informal employ	161 (24.5)	54 (9.7)	154 (28.1)	369 (20.9)	130 (31.0)	132 (22.6)	166 (30.1)	428 (27.5)
No work, receive money from others	144 (21.9)	27 (4.9)	205 (37.3)	376 (21.3)	36 (8.6)	17 (2.9)	146 (26.5)	199 (12.8)
Other	7 (1.1)	16 (2.9)	0	23 (1.3)	2 (0.5)	24 (4.1)	1 (0.2)	27 (1.7)
Missing	1	0	0	1	1	1	1	3

The women in Zimbabwe were older (mean age 36.2 years), while those in Eswatini were younger (mean age 34.7 years). While men in Eswatini were the youngest participants, with a mean age of 33.9 years, Ugandan men were the oldest (mean age 39.8 years). The Zimbabwean women and men had higher levels of education, at 66% and 79%, respectively, having attained secondary and above education. The Ugandan participants had the lowest education, at 20% of the women and 35% of men having secondary or more education. In Zimbabwe and Eswatini, participants tended to be Apostolic or Pentecostal Christian faiths, while the majority of Ugandan participants held more traditional Christian beliefs identifying as Protestant (Anglican) and Roman Catholic. Of note however, was that over 20% of Zimbabwean men reported having no religion.

Over 75% of the participants were long-term community residents (> 5 years). Around one-third of Zimbabwean participants, 32% of women and 39% of men, reported that they had been away from home for > 1 month in the past 12 months, suggesting that they may have migrated for work. Over 75% of the Zimbabwean and Ugandan participants reported being married or living as married, as compared with 58% of women and 42% of men in Eswatini. Participants in Eswatini and Uganda came from larger households, (median household size 6). Among the Ugandan participants, 77% of women and 57% of men were engaged in farming as their main source of income. Only 31% of men and 25% of women in Eswatini farmed, while 37% of the women and 27% of the men received remittances.

Except for the gender equity score in arm 1 for men (p = 0.31), all the interventions led to improvements in scores for both men and women (p < 0.01), as shown in Table 3.

**Table 3:** Indicators scores by study arm and gender, all countries combined

	All countries combined		
	Difference (t-test) between pre-post intervention (95% CI)		P value
Women			
MCH beliefs score			
Arm 1	**1.21 (0.81, 1.60)**		** < 0.001**
Arm 2	**1.72 (1.30, 2.14)**		** < 0.001**
Arm 3	**2.54 (2.11, 2.98)**		** < 0.001**
Stigma score			
Arm 1	**− 1.01 (− 1.46, − 0.55)**		** < 0.001**
Arm 2	**− 1.00 (− 1.43, − 0.56)**		** < 0.001**
Arm 3	**− 1.30 (− 1.72, − 0.88)**		** < 0.001**
Gender equitable score			
Arm 1	**1.27 (0.65, 1.90)**		** < 0.001**
Arm 2	**2.59 (2.00, 3.18)**		** < 0.001**
Arm 3	**1.88 (1.28, 2.47)**		** < 0.001**
Men			
MCH beliefs score			
Arm 2	**1.69 (1.23, 2.16)**		** < 0.001**
Arm 3	**2.93 (2.43, 3.42)**		** < 0.001**
Stigma score			
Arm 1	**− 0.61 (− 1.08, − 0.15)**		**0.010**
Arm 2	**− 1.14 (− 1.62, − 0.65)**		** < 0.001**
Arm 3	**− 1.21 (− 1.69, − 0.74)**		** < 0.001**
Gender equitable score			
Arm 1	0.36 (− 0.34, 1.06)		0.31
Arm 2	**1.73 (1.07, 2.39)**		** < 0.001**
Arm 3	**1.15 (0.49, 1.81)**		**0.0007**

Adjusted for country, age and education

The significance level was defined as *p* < 0.05

The difference in mean indicator score changes by study arms for arms 2 and 3, compared to arm 1, is summarized in Table 4.

**Table 4:** Difference (t-test) in indicator mean score changes pre- vs post-intervention between study arms by gender

	MCH Beliefs Score			HIV Stigma score			Gender-equitable score		
	Arm 2 vs 1	Arm 3 vs 1	Arm 3 vs 2	Arm 2 vs 1	Arm 3 vs 1	Arm 3 vs 2	Arm 2 vs 1	Arm 3 vs 1	Arm 3 vs 2
Women	Diff = 0.52	Diff = 1.34	Diff = 0.82	Diff = 0.008	Diff = − 0.29	Diff = **− **0.30	Diff = 1.32	Diff = 0.60	Diff = **− **0.72
	p = 0.079	p < 0.0001	p = 0.006	p = 0.98	p = 0.35	p = 0.34	p = 0.002	p = 0.16	p = 0.099
Men	Diff = 0.79	Diff = 2.03	Diff = 1.23	Diff = − 0.52	Diff = − 0.60	Diff = **− **0.079	Diff = 1.37	Diff = 0.79	Diff = **− **0.58
	p = 0.018	p < 0.0001	p = 0.0003	p = 0.13	p = 0.083	p = 0.82	p = 0.004	p = 0.11	p = 0.24

Adjusted for country, age and education

For both men and women, at least one of the combined intervention arms (2 and 3) resulted in improved MCH beliefs and GEM scores (p < 0.05), compared with arm 1 (community leader engagement intervention only). For both men and women, adding peer groups led to significant improvements in the MCH beliefs score (arm 3 vs. arm 2) (p < 0.05). However, the GEM score did not change significantly with the addition of the peer groups. For both men and women, the community days and peer group interventions (arms 2 and 3) did not lead to significantly greater improvements in the stigma score, when compared with arm 1(community leader engagement only).

When stratifying these results by country, the combined intervention arms (2 and 3) showed score improvements, for both men and women, for two of the three outcomes (Table 5). However, this improvement is mainly seen for mean community MCH scores and mean stigma scores for two out of the three countries. The only country to have significant improvements in mean GEM scores from the combined interventions for both men and women was Uganda (p < 0.01). The combined intervention improved GEM scores significantly among women in Zimbabwe and Eswatini (p < 0.01), but not among the men. However, similar to the combined country analysis, the addition of the peer group intervention in arm 3 led to improvements in mean MCH beliefs scores, compared to arm 2, for most of the groups studied (p < 0.05). However, the peer group intervention did lead to significant improvements in mean stigma scores for most groups across all three countries (p < 0.01).

**Table 5:** Changes in mean indicator scores by study arm, country and gender

Study arms	Women		Men	
	Difference pre-post intervention (95% CI)	p-value	Difference pre-post intervention (95% CI)	p-value
Zimbabwe				
MCH beliefs score				
Study arm 1	2.86 (2.25, 3.47)	** < .00001**	2.60 (1.69, 3.51)	** < 0.0001**
Study arm 2	4.16 (3.52, 4.79)	** < 0.0001**	3.56 (2.71, 4.40)	** < 0.0001**
Study arm 3	4.01 (3.34, 4.68)	** < .00001**	3.78 (2.97, 4.59)	** < 0.0001**
Stigma score				
Study arm 1	− 2.24 (− 2.85, − 1.63)	** < 0.0001**	− 1.44 (− 2.27, − 0.61)	**0.0008**
Study arm 2	− 1.56 (− 2.18, − 0.94)	** < .00001**	− 2.33 (− 3.11, − 1.54)	** < 0.0001**
Study arm 3	− 1.38 (− 2.03, − 0.72)	** < .00001**	− 0.77 (− 1.64, 0.09)	0.0803
GEM score				
Study arm 1	1.28 (0.39, 2.16)	**0.0044**	1.01 (− 0.21, 2.23)	0.1032
Study arm 2	2.13 (1.23, 3.02)	** < 0.0001**	1.83 (0.70, 2.95)	**0.0015**
Study arm 3	0.29 (− 0.62, 1.21)	0.5267	0.89 (− 0.24, 2.04)	0.1228
Uganda				
MCH beliefs score				
Study arm 1	− 0.83 (− 1.49, − 0.17)	**0.0142**	− 0.54 (− 1.24, 0.16)	0.1313
Study arm 2	− 0.21 (− 0.94, 0.52)	0.5731	0.26 (− 0.51, 1.01)	0.5099
Study arm 3	0.88 (0.17, 1.58)	**0.0153**	2.83 (2.08, 3.58)	** < 0.0001**
Stigma score				
Study arm 1	− 0.37 (− 0.98, 0.25)	0.2391	− 0.23 (− 0.82, 0.36)	0.4385
Study arm 2	− 1.49 (− 2.21, − 0.77)	** < 0.0001**	− 1.41 (− 2.07, − 0.76)	** < 0.0001**
Study arm 3	− 2.49 (− 3.26, − 1.74)	** < 0.0001**	− 2.38 (− 3.08, − 1.67)	** < 0.0001**
GEM score				
Study arm 1	− 1.30 (− 2.10, − 0.51)	**0.0014**	− 1.90 (− 2.83, − 0.98)	** < 0.0001**
Study arm 2	2.19 (1.39, 3.00)	** < 0.0001**	1.01 (0.15, 1.87)	**0.0215**
Study arm 3	1.52 (0.70, 2.34)	**0.0003**	1.42 (0.55, 2.29)	**0.0014**
Eswatini				
MCH beliefs score				
Study arm 1	1.16 (0.46, 1.87)	**0.0013**	1.24 (0.47, 1.99)	**0.0015**
Study arm 2	0.59 (− 0.14, 1.33)	0.1137	1.67 (0.91, 2.43)	** < 0.0001**
Study arm 3	1.75 (1.05, 2.44)	** < 0.0001**	1.71 (0.85, 2.58)	**0.0001**
Stigma score				
Study arm 1	0.58 (0.00, 1.18)	**0.0492**	− 0.33 (− 0.95, 0.29)	**0.3001**
Study arm 2	0.60 (− 0.00, 1.21)	**0.0514**	0.26 (− 0.39, 0.93)	0.4337
Study arm 3	− 0.23 (− 0.78, 0.32)	0.4158	− 0.58 (− 1.29, 0.12)	**0.1040**
GEM score				
Study arm 1	2.10 (1.24, 2.96)	** < 0.0001**	1.77 (0.95, 2.59)	** < 0.0001**
Study arm 2	1.31 (0.53, 2.08)	**0.0010**	1.94 (1.07, 2.81)	** < 0.0001**
Study arm 3	1.52 (0.70, 2.34)	**0.0003**	0.59 (− 0.39, 1.59)	0.2386

Adjusted for age and education

The significance level was defined as *p* < 0.05

Women in arm 3 had significantly greater improvements in mean outcomes scores than the women in arm 1 in Zimbabwe and Uganda. For women in Zimbabwe, those in arm 3 had greater increases in mean MCH beliefs score (diff = 1.15, p = 0.012) than those in arm 1. Among women in Uganda, those in arm 3 had a greater increase in mean MCH beliefs score (diff = 1.71, p = 0.0009), greater reduction in mean HIV stigma score (diff =  − 2.129, p < 0.0001), and greater increase in mean gender equitable norms score (diff = 2.741, p < 0.0001) than those in arm 1.

Among the men, the results were more varied. In Uganda, however, men in arm 3 had a significantly greater increase in mean MCH beliefs score (p < 0.0001), greater decrease in mean HIV stigma score (p < 0.0001), and greater increase in mean GEM scores (p < 0.0001) than men in arm 1.

## Discussion

In this rural population, the results show that for both women and men, arm 2 (community leader engagement plus community days), and arm 3 (community leader engagement, community days, and peer groups) led to greater mean score changes than that of arm 1 (community leader engagement only), for at least two of the three outcome scores. These interventions led to the intended changes in MCH beliefs, HIV stigma, and gender equitable norms among women in the countries studied. However, because both men and women were intentionally included in all three interventions, mean score improvements were seen among the men as well. This showed an increase in MCH beliefs, reduction in HIV stigma, and increased gender equitable norms among men. This is a desirable secondary outcome, as men’s increased MCH and PMTCT beliefs and knowledge, reduced HIV stigma, and increased gender equitable norms could lead to more male involvement in MCH and PMTCT issues. Overall, the educational interventions seemed to be effective for all study arms, with greater improvements in outcomes for the cumulative interventions. These educational interventions could potentially reduce the MTCT rate by increasing MCH beliefs, reducing HIV-related stigma, and encouraging gender equitable norms.

Although there were some differences between the country-by-country and combined analysis results, they both showed that the combined interventions (arms 2 and 3) were more effective than the community leader engagement intervention alone (arm 1), for both women and men. Some of the country differences may have been due to the socioeconomic and demographic differences. Due to the setting, the Ugandan population was less educated, and more traditional, while the population in Eswatini was generally younger, and the Zimbabwean population was the most educated.

Unlike the community leader engagement intervention, the community days and peer group interventions were targeted towards individuals in smaller groups. In the community days, individuals were encouraged to test for HIV and get HIV counseling. In the peer groups, individuals were educated about gender-based violence, safe sex practices, ANC, and PMTCT. This suggests that individuals may be more likely to address their health behaviors regarding HIV prevention and PMTCT when given the chance to discuss these issues in small groups. Other studies have also shown the positive effects of small group and peer interventions on health behaviors regarding HIV prevention behavior and attitudes [16–18].

There were several advantages to the design and analysis of this study. The study of the interventions was done in the form of a randomized trial. This study design controlled for confounding factors, such as demographic variables. Furthermore, it minimized the effect of these confounding factors on the exposure and outcome variables. The sample used for the study was representative of the population**.**

In addition, the specific targeting of men as important players in MCH likely accounted for the level of change seen at the community level. Since the interventions were designed to improve MCH knowledge and gender-equitable norms in the community, the study was able to reach a much broader audience than it would have if it had focused only on HIV-positive women and men.

However, there were limitations to this study design as well. Generalization of the results of this study may be limited to only EGPAF-supported regions or districts, since results differed by country and gender. The pre-/post-intervention assessment design may also be prone to confounding by temporal changes that could have occurred between assessments. In addition, the fact that this was a serial cross-sectional study, rather than a longitudinal study, limits the analytical methods that could be used. The low Cronbach’s alpha scores for MCH beliefs, which suggest low reliability of the measures, could be related to the low number of items in the scales, as well as the fact that the measures used were part of a questionnaire that had number of other questions. The t-test was the most optimal option for analyzing the outcome in this study. An advantage of the t-test was that it allowed for a simple comparison of pre- vs. post-intervention changes in scores for each study arm. However, the t-test did not account for potential demographic confounders. To account for this, t-tests were used to compare mean score changes among demographic categories, but generalized linear models were also used to compare mean score changes between study arms. Therefore, having demographic t-tests, as well as generalized linear model analysis, accounted for the limitations of the initial t-tests. The generalized linear model analysis accounted for potential clustering of participants through inclusion of random effects in the model.

## Conclusion

Arm 3, which combined all three interventions and arm 2 (community leader engagement and community days) led to improvements in outcome scores, compared to the community leader engagement intervention along (arm 1). This suggests that a package of community interventions may be most effective in increasing community HIV knowledge and improving gender equitable norms.
